# Exploring potential new floral organ morphogenesis genes of *Arabidopsis thaliana* using systems biology approach

**DOI:** 10.3389/fpls.2015.00829

**Published:** 2015-10-13

**Authors:** Wenchuan Xie, Junfeng Huang, Yang Liu, Jianan Rao, Da Luo, Miao He

**Affiliations:** School of Life Sciences, Sun Yat-Sen UniversityGuangzhou, China

**Keywords:** *Arabidopsis thaliana*, floral pattern formation, systems biology, co-expression, protein-protein interactions

## Abstract

Flowering is one of the important defining features of angiosperms. The initiation of flower development and the formation of different floral organs are the results of the interplays among numerous genes. But until now, just fewer genes have been found linked with flower development. And the functions of lots of genes of *Arabidopsis thaliana* are still unknown. Although, the quartet model successfully simplified the ABCDE model to elaborate the molecular mechanism by introducing protein-protein interactions (PPIs). We still don't know much about several important aspects of flower development. So we need to discriminate even more genes involving in the flower development. In this study, we identified seven differentially modules through integrating the weighted gene co-expression network analysis (WGCNA) and Support Vector Machine (SVM) method to analyze co-expression network and PPIs using the public floral and non-floral expression profiles data of *Arabidopsis thaliana*. Gene set enrichment analysis was used for the functional annotation of the related genes, and some of the hub genes were identified in each module. The potential floral organ morphogenesis genes of two significant modules were integrated with PPI information in order to detail the inherent regulation mechanisms. Finally, the functions of the floral patterning genes were elucidated by combining the PPI and evolutionary information. It was indicated that the sub-networks or complexes, rather than the genes, were the regulation unit of flower development. We found that the most possible potential new genes underlining the floral pattern formation in *A. thaliana* were *FY, CBL2, ZFN3*, and *AT1G77370*; among them, *FY, CBL2* acted as an upstream regulator of *AP2*; *ZFN3* activated the flower primordial determining gene *AP1* and *AP2* by *HY5*/*HYH* gene via photo induction possibly. And *AT1G77370* exhibited similar function in floral morphogenesis, same as *ELF3*. It possibly formed a complex between *RFC3* and *RPS15* in cytoplasm, which regulated *TSO1* and *CPSF160* in the nucleus, to control the floral organ morphogenesis. This process might also be fine tuning by *AT5G53360* in the nucleus.

## Introduction

Flowering is one of the important defining features of angiosperms. Flowering is also the most pivotal stage that interposes vegetative growth stage and fruiting stage during the development in the higher plants. Each flower starts from a small fraction of undifferentiated cell, and develops into a complex pattern structure while different organs precisely occupy different positions. This process, also named as the floral pattern formation, attracts growing attentions in recent years (Bemis et al., 2013).

The transition from vegetative phase to reproductive phase is of great importance for all flowering plants. The hallmark of the reproductive phase is the differentiation of flower. Shoot apical meristem transforms into floral meristem within this early phase. Latterly, floral organ primordial initiates within the floral meristem and rises to the formation of sepal, petal, stamen and carpel. The development of floral organ is controlled by homeotic genes during reproductive phase. In 1991, the ABC model was proposed by Coen and Meyerowitz (1991) to elaborate the classification of homeotic genes, and to explain the mechanisms of how A, B, and C class genes forming the floral organs in the precise positions during flower development. The hypotheses behind the model are: firstly, the genes in each class were required to function in two adjacent whorls to specify organ types; secondly, each floral organ type originated due to function combination of class A, B, and C genes; finally, class A and class C genes were mutually antagonistic. Colombo et.al revealed that the gene *FBP11* determined ovule development (Colombo et al., 1995) soon, and class D genes were added. In addition, by multiple gene mutants, four SEPALLATA genes were found redundantly interacting with ABC genes to specify floral organ identity (Rounsley et al., 1995). The four class genes are all MADS box transcription factors that are widely spreading in sepal, petal, stamen, carpel and ovule. Furthermore, the ABC model was expanded to ABCDE model. The ABCDE model was meticulous but a little more complicated than the previous one. Protein is the function executor of a gene. From this point of view, a quartet model was proposed by Theissen et al., who presumed that the development of a specific floral organ was achieved by the formation of a single protein complex by both ACB transcription factors and SEPALLATA transcription factors (Theissen and Saedler, 2001). The quartet model successfully simplified the ABCDE model by introducing protein-protein interactions (PPIs).

From the early homologous genes cloning, expression to the later large-scale computational mining the regulating relationships among genes, the flower development in *A. thaliana* had been intensely studied (O'Maoileidigh et al., 2014). The differentially expressed genes between mutant and wild-type of *A. thaliana* had been systematic identified by microarray and experimental results alleging, the floral organ-specific genes were spatially limited expression (Wellmer et al., 2004). The flower organ specification gene regulatory network (FOS-GRN) of *A. thaliana* had been modeled and surveyed the characteristics of network signal transduction (Sanchez-Corrales et al., 2010). But, the effects of PPIs have not been fully considered in flower development research. It was found that the functional tetramers were widespread in the MADS domain protein-protein interaction networks (Espinosa-Soto et al., 2014). So, the protein complexes might provide much more additional information in describing flower development process.

Considerable progress has been made in deciphering the molecular mechanisms underlying the formation of flowers in the past years (Krouk et al., 2013). Floral pattern formation is an extremely complex process. The initiation of flower development and the formation of different floral organs are the results of the interplays among numerous genes. But until now, just a few genes have been found linked with flower development. And the functions of lots of genes of *Arabidopsis thaliana* are still unknown. Several important aspects of flower development still remain poorly understood. So we need to discriminate even more genes involving in the flower development. Several lines of investigation must be followed to address these knowledge gaps and to further unravel the structure and composition of the flowering gene network. The regulatory complexes that control gene expression during flower development must be characterized (O'Maoileidigh et al., 2014). In this research, we're going to identify more potential new genes of the flower development using the systems biology approach, for further understanding the sophisticated relationships of gene regulations underlying the floral pattern formation in *A. thaliana*.

## Materials and methods

### Materials

The gene expression data of *A. thaliana* development were obtained from TAIR (Lamesch et al., 2012). Eighteen samples in triplicate of wild type Columbia (Col-0) were collected from different tissues of *A. thaliana*, and split into two groups by their tissue specificities (Table 1). Both floral group and non-floral group contained data from the same period but with different tissues, particularly, with the florescence stage of floral group ranged from 9 to 12.

**Table 1:** **General microarrays information**.

	**Slide name**	**Period**	**Tissue**	**Florescence**
Non-floral group
	ATGE_101	Col-0age(21 days)	Seedling shoot	
	ATGE_22	Col-0age(21 days)	Whole plant	
	ATGE_90	Col-0age(21 days)	Late rosette	
	ATGE_98	Col-0age(21 days)	Root	
	ATGE_100	Col-0age(21 days)	Seedling shoot	
	ATGE_99	Col-0age(21 days)	Root	
	ATGE_26	Col-0age(21+ days)	Cauline leaf	
	ATGE_27	Col-0age(21+ days)	Internode shoot	
	ATGE_28	Col-0age(21+ days)	Node shoot	
Floral group
	ATGE_31	Col-0age(21+ days)	Stage 9 flower	9
	ATGE_32	Col-0age(21+ days)	Stage 10–11 flower	10~11
	ATGE_33	Col-0age(21+ days)	Stage 12 flower	12
	ATGE_34	Col-0age(21+ days)	Sepal	12
	ATGE_35	Col-0age(21+ days)	Petal	12
	ATGE_36	Col-0age(21+ days)	Stamen	12
	ATGE_37	Col-0age(21+ days)	Carpel	12
	ATGE_92	Col-0age(4 weeks)	Stage 12 flower	12
	ATGE_73	Col-0age(6 weeks)	Pollen	12

The PPI data set of *Arabidopsis* was constructed based on the PPI data which validated by biological experiment, the data mainly came from the following public databases: TAIR (Lamesch et al., 2012), BIND (Willis and Hogue, 2006), BioGRID (Chatr-Aryamontri et al., 2013), IntAct (Kerrien et al., 2012), and MINT (Licata et al., 2012) databases.

### Co-expression network analysis

A gene co-expression network was constructed using the weighted gene co-expression network analysis (WGCNA) method, which implemented with the WGCNA package in *R* (Langfelder and Horvath, 2008). In order to analyze the data within the WGCNA framework in the reasonable time and limited hardware resources, the size of the data set was filtered based on Pearson correlation coefficient (*PCC*) between two genes. There were 6337 genes filtered for WGCNA unsigned co-expression network analysis. A soft-thresholding in the interval (1, 40) was computed, and a soft-thresholding power of 14 with a scale-free model that fitting index *R*^2^ > 0.6 was applied to the maximized scale-free topology structure. While the minimum size of 30 members for each module was chosen.

To incorporate external information into the co-expression network, we used the gene significance (*GS*) measures. Gene significance was defined as *GS*_*i*_ = |*cor*(*x*_*i*_, *T*)|, which indicated correlation of a *x*_*i*_ node expression profile to a phenotypic trait *T*, or a binary trait variable across *m* samples (Langfelder and Horvath, 2008). The network hub was defined as highly connected gene within a network that had high intra-modular connectivity. To identify possible highly connected intra-modular hub genes, module membership (*MM*) was applied. Module Membership was also known as eigengene-based connectivity *kME*, that was defined as *kME*_*cor, i*(*q*)_ = *cor*(*x*_*i*_, *E*(*q*)), where *E*(*q*) was the module eigengene of module *q*.

### Protein-protein interaction analysis

A summary of pre-process was applied to the PPI data sets. Firstly, the protein pairs that contained a protein with < 50 amino acids or unknown amino acids were removed. Secondly, All proteins in the data set were aligned using the multiple sequence alignment tool, cd-hit program (Li and Godzik, 2006), the protein pairs with ≥ 40% identity were removed, and the remaining 6505 protein pairs comprised the final positive data set. Although the overwhelming majority of these pairs had <40% pairwise sequence identity to one another, the classifier would possibly be biased to these homologous sequence pairs.

Since the non-interacting protein pairs were not readily available in *Arabidopsis*, one strategy for constructing negative data set was used. It based on such an assumption, if proteins occupying different subcellular localizations did not interact. The subcellular localization information of the proteins in the positive data set was extracted from SUBA3 (http://suba.plantenergy.uwa.edu.au/) (Tanz et al., 2013). The non-interacting pairs were generated by pairing proteins from different subsets. Here, the negative data set based on subcellular localization information was called Psub. The negative data set must meet three requirements: (i) the protein pairs cannot appear in the whole PPI data set of *Arabidopsis*; (ii) the number of negative pairs is equal to that of positive pairs (Pitre et al., 2006; Shen et al., 2007); (iii) the auto covariance (*AC*) algorithm proposed by Guo et al. (2008), are subsequently fed to LIBSVM (Chang and Lin, 2011) to construct a two-class classification model. The RBF (radial basis function) kernel is used in the support vector machines (SVM) model, the cost (*c*), and gamma (γ) parameters are optimized with grid searching, which are set to 5.278 and 0.574 respectively (Supplementary Figure 1). In addition, co-expression-based PPI was constructed by setting an independent co-expression threshold (α) for the module with high *GS*. Two genes, the co-expression value of which is higher than the threshold, are considered to be interacted in their protein level. The threshold α is calculated by the formula (weight_max_-weight_min_)^*^0.6+weight_average_, where weight_max_ indicates the maximum weight value, with the minimum weight_min_ and the average weight_average_.

### Module enrichment analysis

Gene ontology (GO) enrichment in modules was carried out with ClueGO (Bindea et al., 2009) using Cytoscape v.2.8. The hypergeometric test method was applied (*P* < 0.05). Each module was tested for enrichment in terms of the molecular function (MF) and the biology process (BP) categories. Bonferroni correction method was applied to correct the *P*-values for multiple testing. The ClueGO used kappa statistics to link the functional group terms in the network. The functional groups terms were created by iterative merging of initially defined groups, which based on the predetermined kappa score threshold. The kappa score value could initially be adjusted on a positive scale from zero to one, to limit the network connectivity in a customized way. We functionally grouped network with terms as nodes linked that based on their kappa score ≥0.3. The co-expression network and subcellular localization annotation of interesting genes were visualized by Cerebral (Barsky et al., 2007). Only GO terms with corrected *P* < 0.005 were considered to be overrepresented in our analysis.

### Phylogenetic analysis

Sequences of flower development genes of rice (*Oryza sativa*) (Yoshida and Nagato, 2011), snapdragon (*Antirrhinum majus*) (Hudson et al., 2008), and petunia (*Petunia hybrid*) (Mallona et al., 2010) were retrieved from the literatures. Sequences of flower development genes of *A. thaliana* were selected from the predicted-PPI of brown and magenta modules. Phylogenetic tree was constructed using the alignment-free method to avoid the influence of sequence heterogeneity. The alignment-free method which based on *K*-tuple counting and background subtraction termed a composition vector (*CV*) approach, and the approach was abbreviated as CVTree (Xu and Hao, 2009). *K*-tuple was set to 6, and the resulted tree was visualized by MEGA 5 (Hall, 2013).

## Results

### Modules organization and gene set enrichment analysis

**Figure 1:**
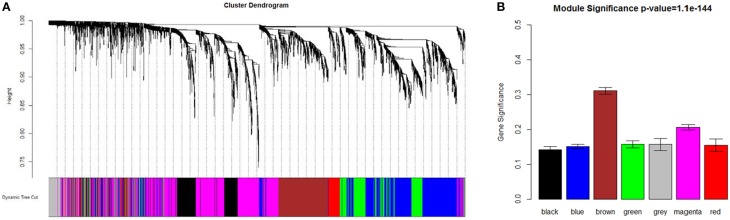
**Network analysis dendrogram showing modules identified by WGCNA**. **(A)** Dendrogram plot with color annotation. **(B)** Module significance.

As shown in Figure 1, a weighted co-expression network with scale-free topology that composed with seven modules of *Arabidopsis* genes was obtained. WGCNA assigned to each module a unique color label that was used as specific module identifier below. The largest module (“magenta”) contained 1333 genes; the least module (“red”) contained 158 genes. Almost 177 probesets were not grouped into any above modules, so they were added to the “gray” module that represented poorly connected genes.

Gene set enrichment analysis of GO terms within module was conducted to provide a biological interpretation for the constructed gene networks (Table 2 and Supplementary Tables 1–6). The magenta module had an over-representation of BP terms related to negative regulation of flower development (*P* = 1.16E-6). Floral organ development (*P* = 2.06E-03) and nuclear-transcribed mRNA catabolic process (*P* = 9.31E-05) were notably enriched in black module. GO terms that included development of floral whorl, carpel and ovule were enriched in blue module (*P* = 1.07E-4). GO terms of far red light respond (*P* = 4.19E-19) and NADPH regeneration (*P* = 1.22E-11) were significantly enriched in green module. Abscisic acid stimulus respond (*P* = 5.12E-06), photomorphogenesis regulation (*P* = 3.09E-04) and interphase of mitotic cell cycle (*P* = 6.37E-25) were notably in brown network. The red module was enriched for genes in regulation of actin filament depolymerization (*P* = 3.75E-04) and the jasmonic acid metabolic process (*P* = 1.87E-03). Hormone-mediated signaling pathway (*P* = 2.42E-6), photomorphogenesis regulation (*P* = 3.09E-04) and RNA splicing (*P* = 3.84E-10) were overrepresented in the magenta and black module.

**Table 2:** **Representative GOTerms in each module identified by ClueGO**.

**Module**	**GOTerm**	**Gene numbers**	***P*-Value**
Black	Hormone-mediated signaling pathway	54	5.64E-09
	Protein glycosylation	23	1.13E-07
	DNA metabolic process	47	1.58E-06
	Cellular response to abscisic acid stimulus	25	5.12E-06
	Nuclear-transcribed mRNA catabolic process	14	9.31E-05
	Regulation of photomorphogenesis	7	3.09E-04
	Positive regulation of signal transduction	7	1.20E-03
	Floral organ development	34	2.06E-03
	Primary shoot apical meristem specification	8	4.53E-03
Blue	Protein targeting to chloroplast	30	1.40E-19
	Isopentenyl diphosphate biosynthetic process	53	2.30E-18
	Plastid membrane organization	44	9.97E-15
	RNA processing	79	3.18E-05
	Hormone-mediated signaling pathway	69	1.01E-03
Brown	Proteasome assembly	52	4.37E-32
	RNA methylation	48	1.02E-27
	Proteolysis involved in cellular protein catabolic process	72	1.84E-27
	Nucleotide biosynthetic process	55	2.33E-25
	Interphase of mitotic cell cycle	45	6.37E-25
	Chromatin organization	70	1.12E-22
	G2 phase of mitotic cell cycle	31	3.12E-19
	Ribonucleotide metabolic process	40	3.52E-17
	Protein import	49	4.46E-15
	RNA metabolic process	156	2.87E-09
	Regulation of gene expression, epigenetic	46	2.37E-07
	mRNA splicing, via spliceosome	18	1.46E-06
	DNA replication initiation	13	5.05E-05
	Phyllome development	37	1.73E-04
	tRNA aminoacylation for protein translation	10	7.62E-04
Green	Photosystem II assembly	39	1.29E-21
	Response to far red light	29	4.19E-19
	Cysteine metabolic process	33	1.63E-13
	NADPH regeneration	29	1.22E-11
	Cellular ion homeostasis	22	1.41E-06
	S-glycoside biosynthetic process	19	1.54E-04
	Regulation of photosynthesis, light reaction	5	6.91E-03
Magenta	Hormone-mediated signaling pathway	122	8.17E-16
	Cell morphogenesis involved in differentiation	78	1.23E-13
	Unidimensional cell growth	80	6.97E-11
	Proteolysis involved in cellular protein catabolic process	68	4.88E-08
	RNA splicing	49	1.94E-07
	Negative regulation of post-embryonic development	22	1.18E-05
	Regulation of cellular macromolecule biosynthetic process	199	3.20E-05
	Regulation of anthocyanin metabolic process	14	1.62E-04
	Photomorphogenesis	37	1.23E-03
	Vegetative to reproductive phase transition of meristem	56	3.93E-03
Red	Regulation of actin filament depolymerization	3	3.75E-04
	Jasmonic acid metabolic process	8	1.87E-03

Each module was filtered to identify the top hub proteins relative to desired criteria using measures, such as intra-modular connectivity (*kME*) and gene significance (*GS*). The Brown module scored the highest among the differentially co-expressed gene modules, followed by the magenta module (Supplementary Figure 2). Multiple genes in the brown module, i.e., *AT1G13030, AT3G09630, AT3G23940, AT4G28450, AT5G07090, AT5G47210, ATARCA, ATG2, CARA, EIF2-GAMMA, GYRA, HD2B, NDPK1, NOP56, NUC-L1, PUR5*, and *TOM40*, were essential factors during the pyrimidine metabolic process. *AT5G38895* and *EIN3* were the factors within reactive oxygen species metabolic process. AT3G14390, also known as diaminopimelate decarboxylase 1, was the hub protein in the brown network. In the magenta module, AHP3, EIN2, ERS1, KEG, PGGT-I, PIF4, RGS1, and RHA2A participated regulations in the signaling pathway. ELF3, GSTU19, HY5, JAR1, PIF4, PKS1, and RD2 were involved in far red light stimulate response.

### PPIs in brown module

Brown module scored the highest among the differentially co-expressed gene modules (*GS* = 0.3109, Figure 1B). The functional annotation showed that this module was enriched in post-embryonic organ morphogenesis, flower organ development and morphogenesis (Supplementary Table 3), which suggested a very important relationship with floral patterning.

**Figure 2:**
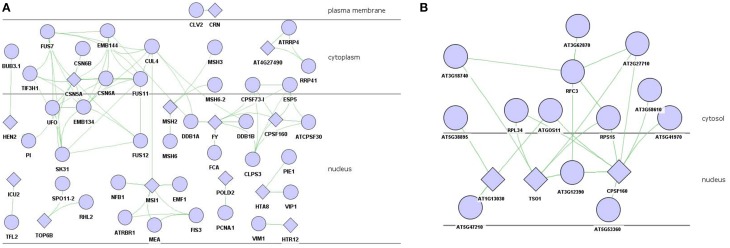
**PPI network of floral organ morphogenesis in brown module. (A)** experiment-PPI. **(B)** predicted-PPI. Rhombus: functional enriched proteins in this module to be concerned.

There were 24 proteins, including FY, EGL3, CRN, CSN5A that involved in floral organ morphogenesis (*P* = 3.18E-04) and also in other floral development process, which were mapped to the experimental PPI databases described above, there were 13 proteins which formed a sub-network (Figure 2A). As the hub protein within the sub-network, CSN5A interacted with FUS7 (COP9), CSN6B, CSN6A, FUS11, FUS12, PI, EMB144 (FUS9), EMB134 (COP8), TIF3H1, and SK31 (FUS6) to form the COP9 signalosome (CSN) complex.

The experimental validated PPIs might present absence in certain interactions. To gain more information, the co-expression value between these 24 proteins and other proteins in the brown module were calculated and filtered with the threshold α setting to 0.08. There were 81 proteins that were selected as highly co-expressed and submitted to the SVM model to predict possible interactions. The interaction results were further filtered to preserve those PPIs with the same subcellular localization. Two proteins who localized in nucleus, i.e., TSO1 and CPSF160, interacted with RPL34, RPS15, AT2G27710, and AT3G12390 (Figure 2B).

### PPIs in magenta module

Genes participated in negative regulation of flower development were found in magenta module, which was the second import module based on gene significance score (Figure 1B). There were 104 genes involved in the flower development (*P* = 3.08E-04) which attracted special attention, including class A genes *AP1* and *AP2*, class B gene *PI*. *AP1/AP2* controlled sepal's development, while *PI* regulated petals development, all of which belonged to the first two stages among floral organ formation.

**Figure 3:**
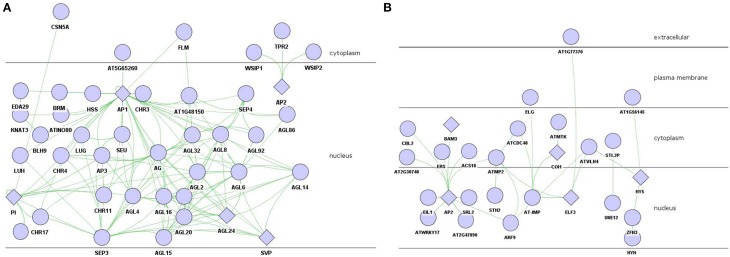
**PPI network of flower development regulation in magenta module. (A)** AP1/AP2/PI involved experiment-PPI. **(B)** predicted-PPI. Rhombus: functional enriched proteins in this module to be concerned.

To decrease the level of complexity, sub-network including *AP1, AP2*, and *PI* was extracted from the 104-genes-based experimental PPIs for further investigation (Figure 3A). AP1, which interacted with AP3, AG, SEU, LUG, SEP3, SEP4, PI, SVP, and AGL, was the hub protein of the sub-experimental PPI. WSIP1, WSIP2, and TPR2 were the interaction partners of AP2 protein.

The predicted-PPI of flowering development in magenta module was constructed similarly as it did in brown module with 101 proteins filter by setting threshold α to 0.16. AP2 was the hub protein in this predicted-PPI which interacted with 10 proteins including CBL2, ERS, SRL2, etc. *AP2*, one of the MADS box transcription factor which belonged to class A, collaborated with *AP1* to regulate the development of sepal and petal.

## Discussions and conclusions

### Modules organization and gene set enrichment analysis

It is always the problem to validate the results from the computational methods. The common cross validation methods are literature retrieval in biological research. We can obtain partial information about the functions of the genes or proteins from the literatures to support our predictions.

It was confirmed by literature retrieval that the early flowering 3 (*ELF3*) of *Arabidopsis* was responsible for generation of circadian rhythm as well as for regulation of photoperiodic flowering (Zhao et al., 2012). The mutation of *ELF3* led to arrhythmic circadian output in continuous light (Covington et al., 2001; Kolmos et al., 2011) and late flowering (Zhao et al., 2012). The membrane-associated progesterone binding protein 2 (ATMP2) was the hub protein in the module based on the *MM*, and took parts in both negative regulation of cellular process and indoleacetic acid biosynthetic process (Kao et al., 2005).

### Potential floral organ morphogenesis genes in brown module

CNS was a conserved protein complex that interacted with CDD complex and covered in the ubiquitin-proteasome pathway, so as to orchestrate the repression of photomorphogenesis (Chen et al., 2006; Nezames and Deng, 2012). The F-box protein, named as Unusual Floral Organs (UFO), also interacted with CSN5A, and participated in flower development of *Arabidopsis* (Wang et al., 2003). Mutation of *UFO* leaded to dramatic changes in floral-organ type (Hepworth et al., 2006). Chae et al. (2008) showed that the UFO, acting as a DNA-associated transcriptional co-factor, was physically interacting with LFY transcription factor to active *AP3* expression in developing petal, stamen primordial and controlling class B and C genes in floral organ formation.

*TSO1* regulated directional processes in cells during floral organogenesis (Hauser et al., 1998). It encoded a floral-specific cell division component, but its function was redundant in non-floral tissue (Liu et al., 1997). This study showed that mutation of *TSO1* displayed defects in cell division of floral meristem cell which including partially formed cell walls and increased DNA ploidy (Liu et al., 1997). *CPSF160*, a subunit of the cleavage and polyadenylation specificity factor (*CPSF*), was an important component of mRNA 3′- end processing apparatus in *Arabidopsis* (Xu et al., 2006). *CPSF* was physically associated with the flowering time regulator *FY* (Herr et al., 2006). It recruited FCA to control *FLC* mRNA expression to affect flowering time (Simpson et al., 2004). The replication factor C subunit 3 (*RFC3*) was high homology to *RFC3* in yeast and other eukaryotic species, functioning in cell replication, proliferation, DNA replication and damage repair (Xia et al., 2009). Genetic research showed that *RFC3* mutation accounts for smaller leaf blades and flower petals, implying that it had cell replication and proliferation functions (Xia et al., 2009), and played an essential role in DNA replication and damage repair (Mossi and Hübscher, 1998). The function of chloroplast ribosomal protein S15 (RPS15) was beyond research, but recent results showed that the replication factor and ribosomal protein might jointly participate in protein synthesis (Daijiro et al., 2014). Thus, we proposed that RFC3 formed a complex with RPS15 in cytoplasmic, and then transported into nucleus, regulating the mRNA expression of *TSO1* and *CPSF160*, further to control the floral organ morphogenesis based on the predicted PPIs. This process might also fine tuning by *AT3G12390* and *AT5G53360* in the nucleus.

### Potential floral organ morphogenesis genes in magenta module

Most of the *AP1* partners belong to the MADS-box family, which are the generally transcription factors (Shore and Sharrocks, 1995) to control all major aspects of development (Becker and Theissen, 2003), and to determine floral organ identity (Ng and Yanofsky, 2001) or flowering time (Michaels and Amasino, 1999) in plant. The MADS-box protein SVP interacted with AP1, SEP3, AGL6 and many other proteins, was a negative regulator of the floral transition (Hartmann et al., 2000). Another MADS-box gene, *FLC*, was also known to repress flowering (Sheldon et al., 1999). SVP consistently interacted with FLC to form a functional heterodimer, and associated with the promoter regions of flowering time regulator FT and SCO1 to repress flowering (Li et al., 2008). Over-expression of *SVP* and/or *FLC* dimerization led to precocious flowering and abnormal floral organ development (Li et al., 2008). *SEP3*, a member of the class E genes, activated class B and C gene expression in stage 3 floral meristem. Class B and C genes did not express because *SEP3* was repressed by *SVP* in floral meristem before late stage 2. This process was reversed by *AP1* through the repression of *SVP*, so as to derepress *SEP3* and *LFY* to activate the genes expression of these two classes in the early stage 3 (Liu et al., 2009).

The antagonistic interaction between class A and class C genes was triggered by *AP2* through negatively regulating *AG*—the C class gene (Krogan et al., 2012). *TPR2* also involved in this process as a binding partner of *AP2* (Figure 3A) (Krogan et al., 2012). *ERS* (ethylene response sensor), a gene in *A. thaliana* ethylene hormone-response pathway, was strongly expressed in young floral primordia and floral organ primordial (Hua et al., 1998). The predicted interaction with AP2 suggested that it might regulate *AP2* in the early stage of flower development. The F-box protein COI1, a critical component of the jasmonate receptor, was also noteworthy. Jasmonates modulate numerous genes expression and mediate responded to stress-related growth inhibition, wounding and pollen development (Devoto et al., 2002; Gfeller et al., 2010). *COI1* mutant was insensitive to methyl jasmonate, and was male sterile due to abnormal pollen production (Xie et al., 1998). Yeast two-hybrid assay showed that the flowering protein terminal flower 2 (TFL2) was associated with the potential transporter AT-IMP (Arabidopsis Interactome Mapping, 2011). *TFL2* had a repressive function in jasmonate signaling, and localized preferentially to euchromatic regions instead of heterochromatic chromocenters (Valdés et al., 2012). COI1 was predicted to associate with AT-IMP in predicted-PPI. We proposed that while *COI1* responded to jasmonate stimulate, AT-IMP was active and transferred the signal to TFL2 to make it engaging in flower development process.

### Functional inference of vital genes in flower development

Above studies showed that, on one hand, the flower development was the complex biological process that multiple genes/proteins involved. The research on gene regulatory network had achieved profound progresses in *Arabidopsis* and other model plant (Azpeitia et al., 2014; O'Maoileidigh et al., 2014). Gene function was directly correlated to specific protein and therefore to its interaction partners. Previous analysis elaborated proteins' role through co-expression clustering and the function of its interaction partners. On the other hand, it was widely accepted that the revolutionary related proteins tended to perform similar function (Ranea et al., 2007; Engelhardt et al., 2011). Thus, we further investigated the evolutionary relationships of flower development genes, which selected from the experiment-PPI/predicted-PPI in brown and magenta module of *A. thaliana* as well as those from rice, snapdragon and petunia that belonged to class A/B/C/D/E genes.

**Figure 4:**
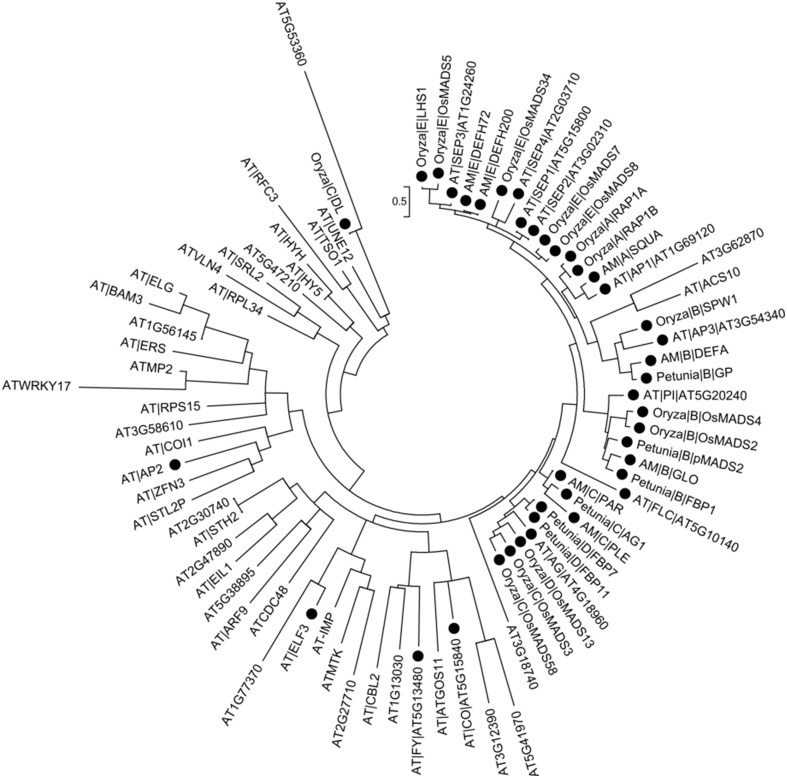
**Phylogenetic analysis of flower development genes**. Solid circles in the figure represent known flower development genes. *Arabidopsis* genes are denoted by AT|symbol|AGI or AGI. Other species genes are denoted by Species|Class|Symbol. “Species” abbreviation: AT, *A. thaliana;* Oryzac, *Oryza sativa;* AM, *Antirrhinum majus;* Petunia, *Petunia hybrida*. “Class” includes A/B/C/D/E. “Symbol” indicates gene symbol.

It was recognized that most of the known proteins in flower development were close to each other in the phylogenetic tree (Figure 4, note by black circle), which suggested that they were evolutionary-related, possibly having the similar biological functions. The result was reasonable as the ABCDE organ identity genes in *Arabidopsis* encoded the MADS-box transcription factors except for the class A gene *AP2* (Figure 4) (Martinez-Castilla and Alvarez-Buylla, 2003). The floral homeotic gene *DROOPING LEAF* (*DL*) in *Oryza*is was distinct from the well-known ABC genes, which had already been defined (Yamaguchi et al., 2004) and also been discussed in phylogenetic tree (Figure 4). It was confirmed that ACS10 closed to class B genes, while in the predicted-PPIs of magenta module, it was predicted to be interacted with AP2 (Figure 3B), which indicated that ACS10 participated in the early stage of floral organ development. It was also found that ACS10 was recorded to express during petal differentiation and expansion stage in TAIR database (https://www.arabidopsis.org/servlets/TairObject?name=AT1G62960&type=locus). CBL2, being clustered with the flowering time regulator FY in the phylogenetic tree, was also predicted that it could associate with *AP2* (Figure 3B). Expression of *CBL2*, being expressed in mature leaves, disappeared during dark treatment while recovering upon illumination, which strongly suggested that it was influential in light-signal transduction (Nozawa et al., 2001). Thus, we proposed that the function of *CBL2* was similar as *FY*, and acted as an upstream regulator of *AP2*. Transcription factor *HY5* controlled light-induced gene expression and targets genes which including light-signaling components and flowering time regulators (Lee et al., 2007). Two genes, *HY5* and *HYH*, were highly similar in *Arabidopsis* (Sibout et al., 2006). The predicted interaction between HY5/HYH and ZFN3 (Figure 3B), and the cluster of ZFN3 and AP2 (Figure 4), indicated that *ZFN3* might be involved in flowering time control. ELF3, AT1G77370, AT2G27710, ATMTK, and AT-IMP were in a similar branch. Few studies had been launched to explore the function of At-IMP, ATMTK, and AT2G27710. However, genetic analysis showed that *ELF3* expressed some functions in early photomorphogenesis (Liu et al., 2001). *AT1G77370*, also named as glutaredoxin-C3, might play a vital role in floral morphogenesis (Wang et al., 2009). Therefore, *ELF3* and *AT1G77370* might exhibit similar function in floral morphogenesis.

## Conclusions and limitations

Floral pattern formation is an extremely complex process. It suffers from the interplay of many different genes. Until now, just a few genes have been found to link with flower development. The functions of lots of genes of *A. thaliana* are still unknown. We need to discriminate even more genes involving in the flower development to better understand the molecular regulation mechanism of the floral pattern formation in *A. thaliana*.

This study aimed to find the possible potential new genes underlining the floral pattern formation in *A. thaliana* by combining the gene expression data, PPIs and phylogenetic information. Results showed that the genes involved in this process could be classified into seven modules with different functions. Furthermore, the brown and magenta modules were significantly correlated with floral organ morphogenesis. By digging into the modules with different types of PPIs information, we endowed each module with real meaning, and it revealed that the PPI networks satisfied the regulatory relationships proposed by ABCDE model.

**Figure 5:**
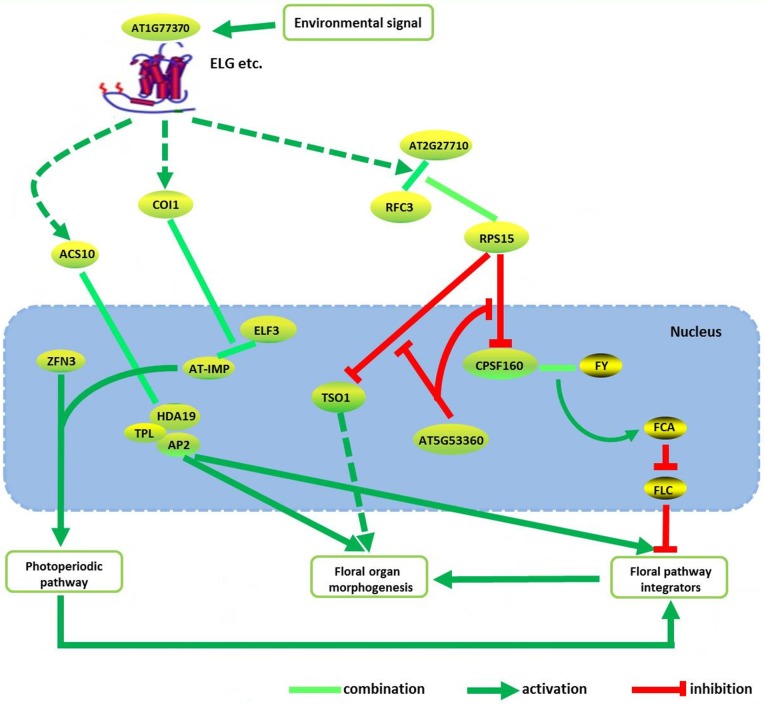
**The integrated pathway of floral pattern formation in ***Arabidopsis thaliana*****. Dotted line indicated the indirect interaction. Some of the proteins/genes combined with AP2 are from the literature (O'Maoileidigh et al., 2014).

It also showed that, the most possible potential new genes of the floral pattern formation in *A. thaliana* were *FY, CBL2, ZFN3*, and *AT1G77370*. *FY* and *CBL2* acted as upstream regulators of *AP2*. *ZFN3* activated the flower primordial determining gene *AP1* and *AP2* by *HY5*/*HYH* gene via photo induction possibly. *AT1G77370* exhibits similar function in floral morphogenesis, same as *ELF3*. *RFC3* forms a complex with *RPS15* in cytoplasmic possibly, to regulate *TSO1* and *CPSF160* in the nucleus, to control the floral organ morphogenesis. This process might also be fine tuning by *AT5G53360* in the nucleus. We inferred a possible pathway to describe the possible molecular regulation mechanism among these genes/proteins of the floral pattern formation in *A. thaliana* by considering some of the previous results (O'Maoileidigh et al., 2014) (see Figure 5).

Generally, the false positives are always existed using *in silico* methods. Novel PPIs and related proteins functions, which are inferred from the module-based PPI networks combining the phylogenetic information, also require to be validated experimentally in the future.

### Conflict of interest statement

The authors declare that the research was conducted in the absence of any commercial or financial relationships that could be construed as a potential conflict of interest.
