# Supplementary material for: Exploring potential new floral organ morphogenesis genes of Arabidopsis thaliana using systems biology approach
Source: Front Plant Sci. 2015 Oct 13;6:829. doi: 10.3389/fpls.2015.00829 (PMC4602108; doi:10.3389/fpls.2015.00829)
Supplement: Supplementary file 1 [file DataSheet1.DOCX]

**Supplementary Table 1. ClueGO analysis of genes in black module**

| Group | GOTerm | Num^1^ | PValue | OverView^2^ |
| --- | --- | --- | --- | --- |
| 0 | nuclear-transcribed mRNA catabolic process | 14 | 9.31E-05 | TRUE |
| 0 | RNA catabolic process | 14 | 2.23E-04 | FALSE |
| 0 | mRNA catabolic process | 14 | 1.04E-04 | FALSE |
| 1 | protein glycosylation | 23 | 1.13E-07 | TRUE |
| 1 | protein N-linked glycosylation | 12 | 1.48E-03 | FALSE |
| 1 | glycoprotein biosynthetic process | 23 | 1.13E-07 | FALSE |
| 2, 4 | regulation of abscisic acid mediated signaling pathway | 8 | 6.07E-03 | FALSE |
| 2, 6 | abscisic acid mediated signaling pathway | 22 | 1.22E-04 | FALSE |
| 2, 6 | hormone-mediated signaling pathway | 54 | 5.64E-09 | TRUE |
| 2, 6 | endoplasmic reticulum unfolded protein response | 17 | 1.09E-03 | FALSE |
| 2, 6 | cellular response to unfolded protein | 17 | 1.23E-03 | FALSE |
| 2, 6 | negative regulation of programmed cell death | 14 | 3.42E-02 | FALSE |
| 2 | negative regulation of abscisic acid mediated signaling pathway | 6 | 1.62E-02 | FALSE |
| 2 | negative regulation of signal transduction | 10 | 3.84E-04 | FALSE |
| 3 | flower development | 47 | 2.09E-03 | FALSE |
| 3 | floral organ development | 34 | 2.06E-03 | TRUE |
| 3, 5 | chromatin organization | 29 | 5.64E-03 | FALSE |
| 3, 5 | regulation of flower development | 23 | 1.61E-02 | FALSE |
| 3, 5 | chromatin modification | 27 | 3.22E-03 | FALSE |
| 3, 5 | covalent chromatin modification | 24 | 1.94E-02 | FALSE |
| 4 | regulation of gibberellic acid mediated signaling pathway | 5 | 6.74E-03 | FALSE |
| 4 | positive regulation of gibberellic acid mediated signaling pathway | 3 | 2.89E-02 | FALSE |
| 4 | raffinose family oligosaccharide biosynthetic process | 4 | 4.28E-02 | FALSE |
| 4, 2 | positive regulation of signal transduction | 7 | 1.20E-03 | TRUE |
| 5 | DNA replication | 20 | 2.43E-02 | FALSE |
| 5 | DNA modification | 18 | 3.47E-04 | FALSE |
| 5 | DNA alkylation | 18 | 3.01E-04 | FALSE |
| 5 | DNA methylation | 18 | 3.01E-04 | FALSE |
| 5 | chromatin silencing | 18 | 1.26E-02 | FALSE |
| 5 | transcription, DNA-dependent | 91 | 1.27E-04 | FALSE |
| 5 | regulation of transcription, DNA-dependent | 87 | 1.87E-05 | FALSE |
| 5 | negative regulation of macromolecule biosynthetic process | 31 | 1.19E-05 | FALSE |
| 5 | negative regulation of gene expression | 40 | 6.87E-06 | FALSE |
| 5 | gene silencing | 27 | 8.73E-03 | FALSE |
| 5 | gene silencing by RNA | 22 | 4.50E-02 | FALSE |
| 5 | negative regulation of cellular biosynthetic process | 31 | 1.82E-05 | FALSE |
| 5 | RNA biosynthetic process | 91 | 1.29E-04 | FALSE |
| 5 | regulation of gene expression, epigenetic | 28 | 2.14E-02 | FALSE |
| 5 | negative regulation of gene expression, epigenetic | 18 | 1.53E-02 | FALSE |
| 5 | negative regulation of transcription, DNA-dependent | 30 | 1.88E-05 | FALSE |
| 5 | negative regulation of nucleobase-containing compound metabolic process | 31 | 1.19E-05 | FALSE |
| 5 | regulation of RNA metabolic process | 87 | 2.12E-05 | FALSE |
| 5 | negative regulation of RNA metabolic process | 30 | 1.88E-05 | FALSE |
| 5 | regulation of cellular macromolecule biosynthetic process | 91 | 1.38E-05 | FALSE |
| 5 | negative regulation of cellular macromolecule biosynthetic process | 31 | 1.19E-05 | FALSE |
| 5 | regulation of RNA biosynthetic process | 87 | 1.87E-05 | FALSE |
| 5, 7 | DNA metabolic process | 47 | 1.58E-06 | TRUE |
| 5, 7 | DNA repair | 19 | 5.13E-03 | FALSE |
| 6 | response to superoxide | 7 | 2.50E-02 | FALSE |
| 6 | protein targeting | 46 | 8.25E-04 | FALSE |
| 6 | protein targeting to membrane | 23 | 2.96E-02 | FALSE |
| 6 | protein targeting to peroxisome | 12 | 1.20E-03 | FALSE |
| 6 | intracellular protein transport | 46 | 4.22E-03 | FALSE |
| 6 | systemic acquired resistance | 30 | 7.84E-04 | FALSE |
| 6 | systemic acquired resistance, salicylic acid mediated signaling pathway | 18 | 4.93E-02 | FALSE |
| 6 | salicylic acid mediated signaling pathway | 29 | 3.01E-05 | FALSE |
| 6 | jasmonic acid mediated signaling pathway | 22 | 1.87E-03 | FALSE |
| 6 | regulation of plant-type hypersensitive response | 23 | 2.24E-02 | FALSE |
| 6 | protein import into peroxisome matrix | 12 | 9.84E-04 | FALSE |
| 6 | positive regulation of cellular biosynthetic process | 32 | 8.55E-03 | FALSE |
| 6 | cellular response to jasmonic acid stimulus | 22 | 1.87E-03 | FALSE |
| 6 | protein localization to peroxisome | 12 | 1.20E-03 | FALSE |
| 6 | establishment of protein localization to peroxisome | 12 | 1.20E-03 | FALSE |
| 6, 2 | cellular response to abscisic acid stimulus | 25 | 5.12E-06 | TRUE |
| 7 | regulation of photomorphogenesis | 7 | 3.09E-04 | TRUE |
| 7 | negative regulation of photomorphogenesis | 5 | 6.74E-03 | FALSE |
| 7 | regulation of response to red or far red light | 7 | 7.34E-04 | FALSE |
| 8 | trichome differentiation | 15 | 1.35E-02 | FALSE |
| 8 | primary shoot apical meristem specification | 8 | 4.53E-03 | TRUE |
| 8 | embryonic meristem development | 9 | 6.35E-03 | FALSE |
| 8 | embryonic meristem initiation | 8 | 1.05E-02 | FALSE |

Num: numbers of gene; OverView: ‘TRUE’ indicates representative GOTerm in relative group

Supplementary Table 2. ClueGO analysis of genes in blue module

| Group | GOTerm | Num | PValue | OverView |
| --- | --- | --- | --- | --- |
| 0 | RNA metabolic process | 186 | 2.86E-03 | FALSE |
| 0 | RNA interference | 23 | 2.23E-02 | FALSE |
| 0 | production of siRNA involved in RNA interference | 19 | 2.93E-02 | FALSE |
| 0 | dsRNA fragmentation | 20 | 1.26E-02 | FALSE |
| 0 | production of miRNAs involved in gene silencing by miRNA | 19 | 1.38E-02 | FALSE |
| 0 | production of small RNA involved in gene silencing by RNA | 20 | 1.26E-02 | FALSE |
| 0, 1 | RNA processing | 79 | 3.18E-05 | TRUE |
| 0, 1 | ncRNA metabolic process | 39 | 4.54E-02 | FALSE |
| 1 | chloroplast organization | 48 | 2.11E-13 | FALSE |
| 1 | plastid membrane organization | 44 | 9.97E-15 | TRUE |
| 1 | chloroplast relocation | 17 | 6.15E-03 | FALSE |
| 1 | thylakoid membrane organization | 44 | 9.97E-15 | FALSE |
| 1 | vegetative to reproductive phase transition of meristem | 50 | 3.78E-05 | FALSE |
| 1 | positive regulation of macromolecule biosynthetic process | 45 | 9.53E-03 | FALSE |
| 1 | positive regulation of cellular biosynthetic process | 50 | 4.63E-02 | FALSE |
| 1 | ovule-producing ovary development | 31 | 2.06E-08 | FALSE |
| 1 | transcription from plastid promoter | 15 | 8.07E-04 | FALSE |
| 1 | positive regulation of nucleobase-containing compound metabolic process | 43 | 2.09E-02 | FALSE |
| 1 | floral whorl development | 45 | 4.18E-02 | FALSE |
| 1 | carpel development | 31 | 1.72E-03 | FALSE |
| 1 | gynoecium development | 32 | 1.47E-03 | FALSE |
| 1 | ovule development | 31 | 1.75E-08 | FALSE |
| 1, 4 | aromatic amino acid family metabolic process | 35 | 2.85E-06 | FALSE |
| 1, 4 | aromatic amino acid family biosynthetic process | 21 | 3.21E-06 | FALSE |
| 1, 4 | iron-sulfur cluster assembly | 25 | 1.64E-09 | FALSE |
| 2 | abscisic acid mediated signaling pathway | 29 | 8.52E-03 | FALSE |
| 2 | hormone-mediated signaling pathway | 69 | 1.01E-03 | TRUE |
| 2 | cellular response to abscisic acid stimulus | 29 | 2.35E-02 | FALSE |
| 3 | protein targeting | 100 | 2.39E-12 | FALSE |
| 3 | protein targeting to vacuole | 28 | 1.70E-09 | FALSE |
| 3 | intracellular protein transport | 102 | 2.40E-11 | FALSE |
| 3 | protein targeting to chloroplast | 30 | 1.40E-19 | TRUE |
| 3 | protein import into chloroplast stroma | 6 | 1.90E-03 | FALSE |
| 3 | intracellular protein transmembrane transport | 9 | 1.85E-05 | FALSE |
| 3 | protein transmembrane transport | 9 | 1.85E-05 | FALSE |
| 3 | establishment of protein localization to organelle | 69 | 8.86E-19 | FALSE |
| 3 | establishment of protein localization to chloroplast | 30 | 1.40E-19 | FALSE |
| 3 | protein localization to chloroplast | 30 | 1.40E-19 | FALSE |
| 3 | protein localization to vacuole | 28 | 1.70E-09 | FALSE |
| 3 | establishment of protein localization to vacuole | 28 | 1.70E-09 | FALSE |
| 4 | sulfur amino acid metabolic process | 40 | 9.25E-05 | FALSE |
| 4 | pyruvate metabolic process | 52 | 7.44E-18 | FALSE |
| 4 | cellular amino acid metabolic process | 85 | 9.37E-11 | FALSE |
| 4 | cysteine metabolic process | 26 | 4.26E-03 | FALSE |
| 4 | glycine metabolic process | 18 | 2.28E-07 | FALSE |
| 4 | glycine catabolic process | 16 | 2.76E-06 | FALSE |
| 4 | unsaturated fatty acid biosynthetic process | 13 | 1.86E-02 | FALSE |
| 4 | nucleoside phosphate metabolic process | 53 | 4.41E-03 | FALSE |
| 4 | porphyrin-containing compound biosynthetic process | 20 | 2.85E-02 | FALSE |
| 4 | isoprenoid biosynthetic process | 63 | 4.64E-10 | FALSE |
| 4 | cellular amino acid biosynthetic process | 62 | 7.30E-09 | FALSE |
| 4 | phospholipid biosynthetic process | 59 | 5.32E-12 | FALSE |
| 4 | cellular amino acid catabolic process | 27 | 4.19E-05 | FALSE |
| 4 | serine family amino acid metabolic process | 39 | 1.46E-07 | FALSE |
| 4 | serine family amino acid biosynthetic process | 26 | 5.92E-03 | FALSE |
| 4 | serine family amino acid catabolic process | 16 | 2.76E-06 | FALSE |
| 4 | lipoate metabolic process | 12 | 3.02E-04 | FALSE |
| 4 | nucleotide metabolic process | 52 | 5.92E-03 | FALSE |
| 4 | isopentenyl diphosphate biosynthetic process | 53 | 2.30E-18 | TRUE |
| 4 | jasmonic acid biosynthetic process | 19 | 2.23E-02 | FALSE |
| 4 | isopentenyl diphosphate biosynthetic process, mevalonate-independent pathway | 52 | 6.11E-18 | FALSE |
| 4 | cysteine biosynthetic process | 26 | 4.26E-03 | FALSE |
| 4 | oxylipin biosynthetic process | 19 | 4.21E-02 | FALSE |
| 4 | monocarboxylic acid metabolic process | 107 | 2.29E-06 | FALSE |
| 4 | unsaturated fatty acid metabolic process | 13 | 2.14E-02 | FALSE |
| 4 | carboxylic acid biosynthetic process | 92 | 8.04E-05 | FALSE |
| 4 | carboxylic acid catabolic process | 39 | 1.68E-02 | FALSE |
| 4 | isopentenyl diphosphate metabolic process | 53 | 2.30E-18 | FALSE |

Num: numbers of gene; OverView: ‘TRUE’ indicates representative GOTerm in relative group

Supplementary Table 3. ClueGO analysis of genes in brown module

| Group | GOTerm | Num | PValue | OverView |
| --- | --- | --- | --- | --- |
| 0, 11, 6 | negative regulation of gene expression | 43 | 5.98E-04 | FALSE |
| 0, 11, 6 | gene silencing | 38 | 5.66E-05 | FALSE |
| 0, 11, 6 | gene silencing by RNA | 32 | 2.89E-04 | FALSE |
| 0, 11, 6 | regulation of gene expression, epigenetic | 46 | 2.37E-07 | TRUE |
| 1, 9 | embryo development ending in seed dormancy | 40 | 4.74E-02 | FALSE |
| 1, 9 | cotyledon development | 11 | 1.51E-02 | FALSE |
| 1, 9 | phyllome development | 37 | 1.73E-04 | TRUE |
| 2, 6, 9 | interphase of mitotic cell cycle | 45 | 6.37E-25 | TRUE |
| 2, 9 | G2 phase of mitotic cell cycle | 31 | 3.12E-19 | TRUE |
| 2, 9 | protein deneddylation | 31 | 4.28E-19 | FALSE |
| 2, 9 | photomorphogenesis | 42 | 3.23E-16 | FALSE |
| 2, 9 | cullin deneddylation | 31 | 3.12E-19 | FALSE |
| 2, 9 | G2 phase | 31 | 3.12E-19 | FALSE |
| 2, 9 | protein modification by small protein removal | 31 | 2.29E-11 | FALSE |
| 2, 9 | protein modification by small protein conjugation or removal | 42 | 7.33E-11 | FALSE |
| 3, 6 | DNA replication initiation | 13 | 5.05E-05 | TRUE |
| 3, 6 | DNA conformation change | 10 | 1.59E-03 | FALSE |
| 4, 14 | glucose metabolic process | 41 | 5.87E-05 | FALSE |
| 4, 14 | gluconeogenesis | 28 | 5.88E-10 | FALSE |
| 4, 14 | proteasomal protein catabolic process | 34 | 2.12E-12 | FALSE |
| 4, 14 | hexose biosynthetic process | 28 | 1.20E-09 | FALSE |
| 4, 14 | proteolysis involved in cellular protein catabolic process | 72 | 1.84E-27 | TRUE |
| 5, 8 | purine nucleotide metabolic process | 17 | 2.11E-05 | FALSE |
| 5, 8 | nucleoside phosphate metabolic process | 67 | 8.85E-17 | FALSE |
| 5, 8 | nucleotide metabolic process | 67 | 3.92E-17 | FALSE |
| 5, 8 | ribonucleotide metabolic process | 40 | 3.52E-17 | TRUE |
| 6 | double-strand break repair via homologous recombination | 12 | 2.14E-04 | FALSE |
| 6 | recombinational repair | 12 | 2.14E-04 | FALSE |
| 6 | DNA repair | 24 | 9.60E-04 | FALSE |
| 6 | double-strand break repair | 17 | 1.61E-05 | FALSE |
| 6 | DNA modification | 24 | 4.92E-06 | FALSE |
| 6 | DNA alkylation | 24 | 4.02E-06 | FALSE |
| 6 | DNA methylation | 24 | 4.02E-06 | FALSE |
| 6 | DNA recombination | 21 | 1.38E-02 | FALSE |
| 6 | chromatin organization | 70 | 1.12E-22 | TRUE |
| 6 | flower development | 70 | 1.07E-07 | FALSE |
| 6 | somatic cell DNA recombination | 7 | 1.38E-02 | FALSE |
| 6 | chromatin silencing by small RNA | 13 | 3.57E-02 | FALSE |
| 6 | histone lysine methylation | 43 | 1.36E-17 | FALSE |
| 6 | floral organ development | 38 | 2.40E-02 | FALSE |
| 6 | floral organ morphogenesis | 24 | 3.18E-04 | FALSE |
| 6 | floral organ formation | 23 | 1.07E-05 | FALSE |
| 6 | post-embryonic organ morphogenesis | 24 | 3.18E-04 | FALSE |
| 6 | organ formation | 24 | 2.44E-05 | FALSE |
| 6 | histone H3-K9 methylation | 28 | 5.93E-09 | FALSE |
| 8 | pyrimidine nucleotide metabolic process | 33 | 3.69E-17 | FALSE |
| 8 | pyrimidine nucleotide biosynthetic process | 33 | 3.69E-17 | FALSE |
| 8 | nucleotide biosynthetic process | 55 | 2.33E-25 | TRUE |
| 8 | pyrimidine ribonucleotide metabolic process | 33 | 2.92E-17 | FALSE |
| 8 | pyrimidine ribonucleotide biosynthetic process | 33 | 2.92E-17 | FALSE |
| 8 | ribonucleotide biosynthetic process | 35 | 7.46E-17 | FALSE |
| 8 | nucleoside phosphate biosynthetic process | 55 | 2.81E-25 | FALSE |
| 10 | RNA methylation | 48 | 1.02E-27 | TRUE |
| 10 | RNA modification | 49 | 4.01E-13 | FALSE |
| 10, 7 | RNA metabolic process | 156 | 2.87E-09 | TRUE |
| 12 | RNA export from nucleus | 14 | 1.19E-05 | FALSE |
| 0, 11, 7 | RNA splicing, via transesterification reactions | 18 | 6.43E-06 | FALSE |
| 0, 11, 7 | RNA splicing, via transesterification reactions with bulged adenosine as nucleophile | 18 | 6.43E-06 | FALSE |
| 0, 11, 7 | mRNA splicing, via spliceosome | 18 | 1.46E-06 | TRUE |
| 0, 11, 7 | mRNA processing | 21 | 2.28E-06 | FALSE |
| 12 | mRNA export from nucleus | 13 | 1.98E-05 | FALSE |
| 12 | protein targeting | 62 | 8.69E-06 | FALSE |
| 12 | protein import into nucleus | 22 | 1.35E-06 | FALSE |
| 12 | protein targeting to mitochondrion | 24 | 1.13E-11 | FALSE |
| 12 | intracellular protein transport | 64 | 1.76E-05 | FALSE |
| 12 | nucleocytoplasmic transport | 31 | 2.47E-10 | FALSE |
| 12 | protein import | 49 | 4.46E-15 | TRUE |
| 12 | protein localization to nucleus | 22 | 2.16E-06 | FALSE |
| 12 | RNA transport | 14 | 1.19E-05 | FALSE |
| 12 | mRNA transport | 13 | 1.98E-05 | FALSE |
| 12 | nuclear export | 15 | 3.33E-06 | FALSE |
| 12 | nuclear import | 22 | 1.70E-06 | FALSE |
| 12 | protein localization to mitochondrion | 24 | 1.13E-11 | FALSE |
| 12 | establishment of protein localization to organelle | 37 | 1.59E-06 | FALSE |
| 12 | establishment of protein localization to mitochondrion | 24 | 1.13E-11 | FALSE |
| 13 | tRNA aminoacylation for protein translation | 10 | 7.62E-04 | TRUE |
| 13 | amino acid activation | 10 | 7.62E-04 | FALSE |
| 13 | tRNA aminoacylation | 10 | 7.62E-04 | FALSE |
| 14 | ubiquitin-dependent protein catabolic process | 63 | 3.35E-26 | FALSE |
| 14 | modification-dependent protein catabolic process | 64 | 1.26E-26 | FALSE |
| 14 | proteasomal ubiquitin-dependent protein catabolic process | 25 | 4.59E-11 | FALSE |
| 14 | proteasome assembly | 52 | 4.37E-32 | TRUE |
| 14 | cellular protein complex assembly | 62 | 8.92E-16 | FALSE |
| 14 | proteasome core complex assembly | 41 | 4.11E-27 | FALSE |

Num: numbers of gene; OverView: ‘TRUE’ indicates representative GOTerm in relative group

Supplementary Table 4. ClueGO analysis of genes in green module

| Group | GOTerm | Num | PValue | OverView |
| --- | --- | --- | --- | --- |
| 0 | leaf morphogenesis | 17 | 3.75E-02 | FALSE |
| 0 | positive regulation of macromolecule biosynthetic process | 30 | 8.55E-03 | FALSE |
| 0 | positive regulation of gene expression | 29 | 1.21E-02 | FALSE |
| 0 | chlorophyll metabolic process | 18 | 2.21E-03 | FALSE |
| 0 | positive regulation of transcription, DNA-dependent | 28 | 2.24E-02 | FALSE |
| 0 | positive regulation of nucleobase-containing compound metabolic process | 29 | 1.21E-02 | FALSE |
| 0 | leaf development | 27 | 1.08E-02 | FALSE |
| 0 | positive regulation of RNA metabolic process | 28 | 2.24E-02 | FALSE |
| 0, 3 | pentose-phosphate shunt | 28 | 7.00E-11 | FALSE |
| 0, 3 | NADP metabolic process | 29 | 1.86E-11 | FALSE |
| 0, 3 | NADPH regeneration | 29 | 1.22E-11 | TRUE |
| 0, 3 | nucleoside phosphate metabolic process | 38 | 3.23E-04 | FALSE |
| 0, 3 | nucleotide metabolic process | 38 | 2.25E-04 | FALSE |
| 0, 3 | chlorophyll biosynthetic process | 12 | 4.16E-02 | FALSE |
| 0, 3 | pyridine nucleotide metabolic process | 29 | 6.18E-11 | FALSE |
| 0, 3 | nicotinamide nucleotide metabolic process | 29 | 3.66E-11 | FALSE |
| 1 | protein phosphorylation | 29 | 3.75E-02 | FALSE |
| 1 | response to high light intensity | 25 | 2.43E-06 | FALSE |
| 1 | response to sucrose stimulus | 24 | 2.46E-06 | FALSE |
| 1 | regulation of proton transport | 25 | 3.01E-17 | FALSE |
| 1 | monovalent inorganic cation transport | 32 | 1.07E-10 | FALSE |
| 1 | proton transport | 25 | 1.10E-13 | FALSE |
| 1 | protein autophosphorylation | 19 | 5.35E-06 | FALSE |
| 2 | S-glycoside biosynthetic process | 19 | 1.54E-04 | TRUE |
| 2 | glycosinolate metabolic process | 20 | 7.29E-04 | FALSE |
| 2 | glycosinolate biosynthetic process | 19 | 1.54E-04 | FALSE |
| 2 | glucosinolate metabolic process | 20 | 7.29E-04 | FALSE |
| 2 | glucosinolate biosynthetic process | 19 | 1.54E-04 | FALSE |
| 3 | maltose metabolic process | 14 | 2.21E-02 | FALSE |
| 3 | glucose metabolic process | 44 | 1.74E-08 | FALSE |
| 3 | glucose catabolic process | 43 | 3.45E-10 | FALSE |
| 3 | PSII associated light-harvesting complex II catabolic process | 8 | 1.22E-04 | FALSE |
| 3 | RNA metabolic process | 116 | 2.53E-03 | FALSE |
| 3 | hexose catabolic process | 43 | 3.99E-10 | FALSE |
| 3 | cellular protein complex assembly | 48 | 2.52E-10 | FALSE |
| 4 | photoinhibition | 4 | 1.15E-02 | FALSE |
| 4 | regulation of photosynthesis, light reaction | 5 | 6.91E-03 | TRUE |
| 4 | negative regulation of photosynthesis, light reaction | 4 | 1.15E-02 | FALSE |
| 5 | sulfur amino acid metabolic process | 34 | 3.39E-08 | FALSE |
| 5 | sulfur amino acid biosynthetic process | 34 | 1.30E-09 | FALSE |
| 5 | MAPK cascade | 29 | 1.72E-09 | FALSE |
| 5 | cellular amino acid metabolic process | 42 | 3.56E-03 | FALSE |
| 5 | cysteine metabolic process | 33 | 1.63E-13 | TRUE |
| 5 | protein targeting | 45 | 2.68E-02 | FALSE |
| 5 | protein targeting to membrane | 38 | 7.72E-09 | FALSE |
| 5 | intracellular protein kinase cascade | 29 | 6.71E-09 | FALSE |
| 5 | cellular amino acid biosynthetic process | 38 | 8.14E-06 | FALSE |
| 5 | serine family amino acid metabolic process | 33 | 1.02E-10 | FALSE |
| 5 | serine family amino acid biosynthetic process | 33 | 2.87E-13 | FALSE |
| 5 | systemic acquired resistance | 38 | 1.57E-06 | FALSE |
| 5 | salicylic acid metabolic process | 24 | 9.44E-06 | FALSE |
| 5 | salicylic acid biosynthetic process | 24 | 3.49E-06 | FALSE |
| 5 | hormone-mediated signaling pathway | 59 | 2.36E-09 | FALSE |
| 5 | systemic acquired resistance, salicylic acid mediated signaling pathway | 32 | 4.09E-09 | FALSE |
| 5 | salicylic acid mediated signaling pathway | 36 | 5.64E-08 | FALSE |
| 5 | jasmonic acid mediated signaling pathway | 35 | 3.31E-10 | FALSE |
| 5 | regulation of hydrogen peroxide metabolic process | 28 | 5.42E-10 | FALSE |
| 5 | regulation of plant-type hypersensitive response | 36 | 6.47E-08 | FALSE |
| 5 | cysteine biosynthetic process | 33 | 1.63E-13 | FALSE |
| 5 | negative regulation of defense response | 33 | 2.20E-09 | FALSE |
| 5 | negative regulation of programmed cell death | 15 | 3.01E-02 | FALSE |
| 5 | regulation of innate immune response | 38 | 1.28E-07 | FALSE |
| 5 | carboxylic acid biosynthetic process | 66 | 8.40E-07 | FALSE |
| 5 | cellular response to jasmonic acid stimulus | 35 | 3.31E-10 | FALSE |
| 6 | cellular ion homeostasis | 22 | 1.41E-06 | TRUE |
| 6 | cellular cation homeostasis | 21 | 1.50E-06 | FALSE |
| 6 | cation homeostasis | 21 | 4.08E-05 | FALSE |
| 6 | divalent metal ion transport | 18 | 6.55E-03 | FALSE |
| 6 | divalent inorganic cation transport | 18 | 1.04E-02 | FALSE |
| 0, 3, 5 | regulation of cellular protein metabolic process | 28 | 2.07E-07 | FALSE |
| 0, 5 | shoot morphogenesis | 26 | 4.13E-02 | FALSE |
| 1, 3 | photosynthetic electron transport in photosystem I | 16 | 6.99E-11 | FALSE |
| 1, 3 | response to red light | 26 | 7.90E-15 | FALSE |
| 1, 3 | response to far red light | 29 | 4.19E-19 | TRUE |
| 1, 3, 5 | rRNA processing | 33 | 1.17E-08 | FALSE |
| 1, 3, 5 | chloroplast organization | 19 | 2.16E-02 | FALSE |
| 1, 3, 5 | chloroplast relocation | 17 | 1.49E-06 | FALSE |
| 1, 3, 5 | rRNA metabolic process | 33 | 1.27E-08 | FALSE |
| 1, 3, 5 | ncRNA processing | 34 | 7.18E-08 | FALSE |
| 1, 3, 5 | ncRNA metabolic process | 36 | 9.44E-07 | FALSE |
| 3, 5 | RNA processing | 44 | 2.64E-02 | FALSE |
| 3, 5 | protein dephosphorylation | 26 | 2.38E-09 | FALSE |
| 3, 5 | plastid membrane organization | 16 | 3.26E-02 | FALSE |
| 3, 5 | thylakoid membrane organization | 16 | 3.26E-02 | FALSE |
| 3, 5 | photosystem II assembly | 39 | 1.29E-21 | TRUE |
| 3, 5 | regulation of phosphate metabolic process | 25 | 8.19E-08 | FALSE |
| 3, 5 | regulation of protein modification process | 25 | 8.19E-08 | FALSE |
| 3, 5 | regulation of dephosphorylation | 25 | 5.87E-11 | FALSE |
| 3, 5 | regulation of protein dephosphorylation | 25 | 2.58E-11 | FALSE |

Num: numbers of gene; OverView: ‘TRUE’ indicates representative GOTerm in relative group

Supplementary Table 5. ClueGO analysis of genes in magenta module

| Group | GOTerm | Num | PValue | OverView |
| --- | --- | --- | --- | --- |
| 0 | abscisic acid mediated signaling pathway | 48 | 2.64E-08 | FALSE |
| 0 | hormone-mediated signaling pathway | 122 | 8.17E-16 | TRUE |
| 0 | regulation of abscisic acid mediated signaling pathway | 14 | 1.21E-03 | FALSE |
| 0 | ethylene mediated signaling pathway | 26 | 1.89E-04 | FALSE |
| 0 | negative regulation of signal transduction | 13 | 4.07E-02 | FALSE |
| 0 | regulation of ethylene mediated signaling pathway | 7 | 9.47E-03 | FALSE |
| 0 | negative regulation of ethylene mediated signaling pathway | 6 | 2.28E-02 | FALSE |
| 0 | regulation of two-component signal transduction system (phosphorelay) | 7 | 9.47E-03 | FALSE |
| 0 | negative regulation of two-component signal transduction system (phosphorelay) | 6 | 2.28E-02 | FALSE |
| 0 | cellular response to abscisic acid stimulus | 48 | 1.95E-07 | FALSE |
| 0 | cellular response to ethylene stimulus | 28 | 2.51E-04 | FALSE |
| 1 | cytokinin mediated signaling pathway | 18 | 1.52E-03 | FALSE |
| 1 | regulation of anthocyanin metabolic process | 14 | 1.62E-04 | TRUE |
| 1 | anthocyanin-containing compound metabolic process | 20 | 1.34E-03 | FALSE |
| 1 | cellular response to cytokinin stimulus | 18 | 1.52E-03 | FALSE |
| 3 | flower development | 104 | 3.08E-04 | FALSE |
| 3 | regulation of flower development | 52 | 2.00E-04 | FALSE |
| 3 | negative regulation of flower development | 14 | 5.37E-04 | FALSE |
| 3 | negative regulation of post-embryonic development | 22 | 1.18E-05 | TRUE |
| 4 | actin filament organization | 38 | 6.83E-10 | FALSE |
| 4 | regulation of actin polymerization or depolymerization | 27 | 5.48E-07 | FALSE |
| 4 | actin polymerization or depolymerization | 29 | 1.35E-07 | FALSE |
| 4 | epidermal cell differentiation | 71 | 1.99E-06 | FALSE |
| 4 | meristem structural organization | 44 | 1.24E-05 | FALSE |
| 4 | meristem initiation | 32 | 6.47E-05 | FALSE |
| 4 | root morphogenesis | 62 | 3.19E-05 | FALSE |
| 4 | trichome differentiation | 38 | 1.76E-07 | FALSE |
| 4 | root epidermal cell differentiation | 52 | 2.54E-04 | FALSE |
| 4 | trichoblast differentiation | 50 | 2.72E-04 | FALSE |
| 4 | primary shoot apical meristem specification | 12 | 2.07E-02 | FALSE |
| 4 | meristem maintenance | 38 | 3.64E-02 | FALSE |
| 4 | trichome morphogenesis | 36 | 1.31E-07 | FALSE |
| 4 | positive regulation of organelle organization | 30 | 9.33E-06 | FALSE |
| 4 | actin cytoskeleton organization | 39 | 1.64E-08 | FALSE |
| 4 | actin filament polymerization | 28 | 2.17E-07 | FALSE |
| 4 | regulation of actin filament length | 27 | 5.48E-07 | FALSE |
| 4 | regulation of actin filament polymerization | 27 | 4.39E-07 | FALSE |
| 4 | positive regulation of actin filament polymerization | 26 | 8.87E-07 | FALSE |
| 4 | positive regulation of protein complex assembly | 26 | 1.38E-06 | FALSE |
| 4 | regulation of protein polymerization | 27 | 1.05E-06 | FALSE |
| 4 | positive regulation of protein polymerization | 26 | 1.38E-06 | FALSE |
| 4 | regulation of actin cytoskeleton organization | 27 | 5.48E-07 | FALSE |
| 4 | regulation of protein complex assembly | 27 | 1.05E-06 | FALSE |
| 4 | actin nucleation | 26 | 8.87E-07 | FALSE |
| 4 | flower morphogenesis | 16 | 6.20E-03 | FALSE |
| 4 | embryonic meristem development | 14 | 2.92E-02 | FALSE |
| 4 | trichoblast maturation | 49 | 3.63E-05 | FALSE |
| 4 | root hair cell differentiation | 49 | 3.63E-05 | FALSE |
| 4 | protein polymerization | 28 | 1.19E-06 | FALSE |
| 4 | regulation of cytoskeleton organization | 27 | 2.39E-06 | FALSE |
| 4 | positive regulation of cytoskeleton organization | 26 | 2.11E-06 | FALSE |
| 4 | embryonic meristem initiation | 13 | 1.58E-02 | FALSE |
| 5 | photoperiodism, flowering | 25 | 3.70E-02 | FALSE |
| 6 | transcription, DNA-dependent | 202 | 3.06E-04 | FALSE |
| 6 | regulation of transcription, DNA-dependent | 189 | 5.75E-05 | FALSE |
| 6 | positive regulation of cellular biosynthetic process | 70 | 1.01E-03 | FALSE |
| 6 | RNA biosynthetic process | 202 | 3.15E-04 | FALSE |
| 6 | positive regulation of nucleobase-containing compound metabolic process | 53 | 4.75E-02 | FALSE |
| 6 | regulation of RNA metabolic process | 190 | 4.47E-05 | FALSE |
| 6 | regulation of cellular macromolecule biosynthetic process | 199 | 3.20E-05 | TRUE |
| 6 | regulation of RNA biosynthetic process | 189 | 5.75E-05 | FALSE |
| 7 | glucose metabolic process | 71 | 2.73E-06 | FALSE |
| 7 | glucose catabolic process | 56 | 1.29E-03 | FALSE |
| 7 | gluconeogenesis | 36 | 2.56E-07 | FALSE |
| 7 | glycolysis | 37 | 6.59E-06 | FALSE |
| 7 | hexose biosynthetic process | 37 | 1.54E-07 | FALSE |
| 7 | hexose catabolic process | 56 | 1.47E-03 | FALSE |
| 8 | RNA splicing, via endonucleolytic cleavage and ligation | 29 | 2.16E-05 | FALSE |
| 8 | methionine metabolic process | 23 | 1.01E-04 | FALSE |
| 8 | RNA splicing | 49 | 1.94E-07 | TRUE |
| 8 | aspartate family amino acid metabolic process | 24 | 6.99E-03 | FALSE |
| 8 | aspartate family amino acid biosynthetic process | 21 | 1.02E-02 | FALSE |
| 8 | methionine biosynthetic process | 21 | 6.79E-04 | FALSE |
| 9 | photomorphogenesis | 37 | 1.23E-03 | TRUE |
| 9 | protein ubiquitination | 28 | 4.05E-02 | FALSE |
| 9 | protein modification by small protein conjugation | 31 | 1.38E-02 | FALSE |
| 2, 4 | cell morphogenesis involved in differentiation | 78 | 1.23E-13 | TRUE |
| 4, 2 | unidimensional cell growth | 80 | 6.97E-11 | TRUE |
| 2, 4 | pollen tube growth | 42 | 5.90E-06 | FALSE |
| 2, 4 | cell tip growth | 56 | 1.25E-07 | FALSE |
| 2, 4, 7 | proteasome assembly | 32 | 1.61E-04 | FALSE |
| 2, 7 | proteasomal protein catabolic process | 35 | 1.37E-04 | FALSE |
| 2, 7 | regulation of cell morphogenesis | 20 | 7.99E-04 | FALSE |
| 2, 7 | DNA endoreduplication | 21 | 1.51E-02 | FALSE |
| 2, 7 | regulation of unidimensional cell growth | 19 | 1.11E-03 | FALSE |
| 4, 7 | ubiquitin-dependent protein catabolic process | 51 | 6.81E-05 | FALSE |
| 4, 7 | modification-dependent protein catabolic process | 51 | 1.15E-04 | FALSE |
| 4, 7 | cellular protein complex assembly | 72 | 3.31E-06 | FALSE |
| 7, 4 | proteolysis involved in cellular protein catabolic process | 68 | 4.88E-08 | TRUE |
| 4, 7 | proteasome core complex assembly | 25 | 4.25E-04 | FALSE |
| 5, 9 | vegetative to reproductive phase transition of meristem | 56 | 3.93E-03 | TRUE |
| 5, 9 | protein modification by small protein conjugation or removal | 44 | 1.19E-02 | FALSE |
| 6, 8 | RNA processing | 89 | 1.78E-02 | FALSE |
| 6, 8 | RNA metabolic process | 251 | 2.53E-04 | FALSE |

Num: numbers of gene; OverView: ‘TRUE’ indicates representative GOTerm in relative group

Supplementary Table 6. ClueGO analysis of genes in red module

| Group | GOTerm | Num | PValue | OverView |
| --- | --- | --- | --- | --- |
| 0 | jasmonic acid metabolic process | 8 | 1.87E-03 | TRUE |
| 0 | jasmonic acid biosynthetic process | 6 | 2.85E-02 | FALSE |
| 1 | negative regulation of organelle organization | 3 | 5.60E-03 | FALSE |
| 1 | actin filament depolymerization | 3 | 1.01E-03 | FALSE |
| 1 | regulation of actin filament depolymerization | 3 | 3.75E-04 | TRUE |
| 1 | negative regulation of actin filament depolymerization | 2 | 1.24E-02 | FALSE |
| 1 | negative regulation of actin filament polymerization | 2 | 1.24E-02 | FALSE |
| 1 | negative regulation of protein complex assembly | 2 | 2.80E-02 | FALSE |
| 0 | oxylipin metabolic process | 8 | 2.47E-03 | FALSE |
| 0 | oxylipin biosynthetic process | 6 | 3.43E-02 | FALSE |
| 1 | negative regulation of protein polymerization | 2 | 2.80E-02 | FALSE |
| 1 | negative regulation of protein complex disassembly | 2 | 1.24E-02 | FALSE |
| 1 | regulation of protein complex disassembly | 3 | 1.01E-03 | FALSE |
| 1 | cellular protein complex disassembly | 3 | 1.86E-02 | FALSE |
| 1 | protein depolymerization | 3 | 2.04E-03 | FALSE |
| 1 | negative regulation of cytoskeleton organization | 2 | 2.80E-02 | FALSE |
| 1 | actin filament capping | 2 | 1.24E-02 | FALSE |

Num: numbers of gene; OverView: ‘TRUE’ indicates representative GOTerm in relative group
