# Supplementary figures and images for: Exploring potential new floral organ morphogenesis genes of Arabidopsis thaliana using systems biology approach
Source: Front Plant Sci. 2015 Oct 13;6:829. doi: 10.3389/fpls.2015.00829 (PMC4602108; doi:10.3389/fpls.2015.00829)

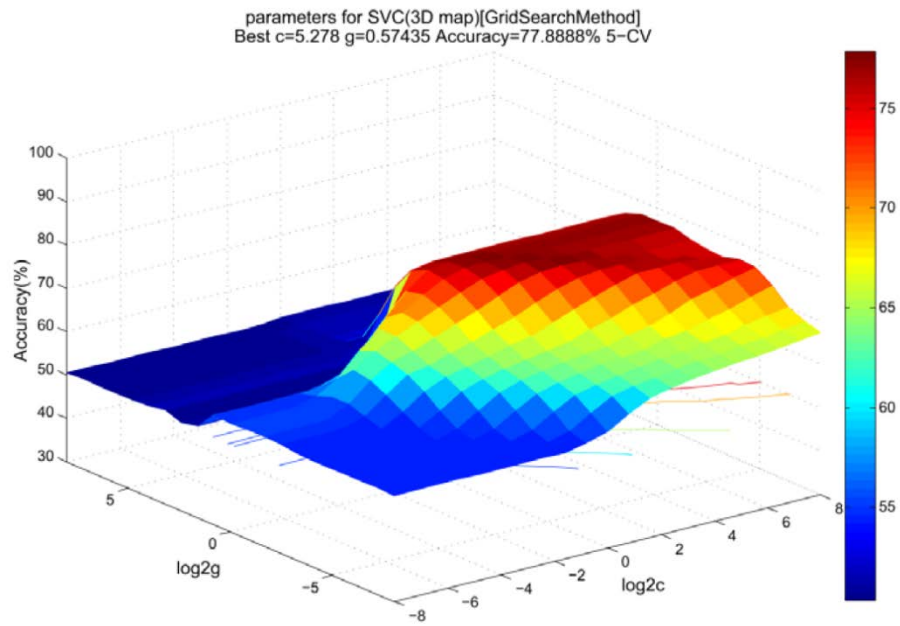

**Supplementary Fig 1. Grid search for SVM-based PPI model parameters identification**

Supplement: Supplementary file 2 [file Image1.PDF]

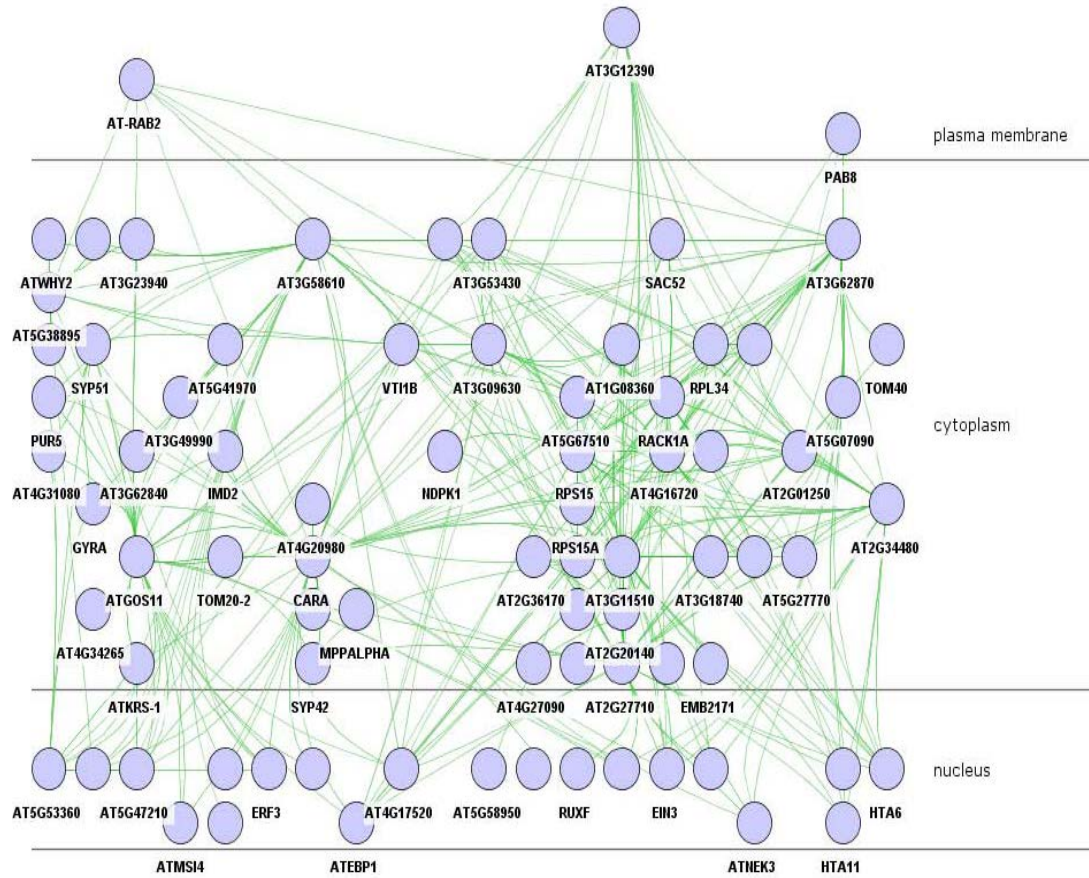

Brown module

Supplement: Supplementary file 3 [file Image2.PDF]
